# Augmentation of response to nab-paclitaxel by inhibition of insulin-like growth factor (IGF) signaling in preclinical pancreatic cancer models

**DOI:** 10.18632/oncotarget.9029

**Published:** 2016-04-26

**Authors:** Niranjan Awasthi, Emily Scire, Sheena Monahan, Meghan Grojean, Eric Zhang, Margaret A. Schwarz, Roderich E. Schwarz

**Affiliations:** ^1^ Department of Surgery, Indiana University School of Medicine, South Bend, IN, USA; ^2^ Department of Chemistry and Biochemistry, University of Notre Dame, Notre Dame, IN, USA; ^3^ Department of Psychology, University of Notre Dame, Notre Dame, IN, USA; ^4^ Department of Pediatrics, Indiana University School of Medicine, South Bend, IN, USA; ^5^ Harper Cancer Research Institute, University of Notre Dame, Notre Dame, IN, USA; ^6^ Indiana University Health Goshen Center for Cancer Care, Goshen, IN, USA

**Keywords:** pancreatic cancer, nab-paclitaxel, IGF-1R, BMS-754807, combination therapy

## Abstract

Nab-paclitaxel has recently shown greater efficacy in pancreatic ductal adenocarcinoma (PDAC). Insulin like growth factor (IGF) signaling proteins are frequently overexpressed in PDAC and correlate with aggressive tumor phenotype and poor prognosis. We evaluated the improvement in nab-paclitaxel response by addition of BMS-754807, a small molecule inhibitor of IGF-1R/IR signaling, in preclinical PDAC models. In subcutaneous xenografts using AsPC-1 cells, average net tumor growth in different therapy groups was 248.3 mm^3^ in controls, 42.4 mm^3^ after nab-paclitaxel (*p* = 0.002), 93.3 mm^3^ after BMS-754807 (*p* = 0.01) and 1.9 mm^3^ after nab-paclitaxel plus BMS-754807 (*p* = 0.0002). In subcutaneous xenografts using Panc-1 cells, average net tumor growth in different therapy groups was: 294.3 mm^3^ in controls, 23.1 mm^3^ after nab-paclitaxel (*p* = 0.002), 118.2 mm^3^ after BMS-754807 (*p* = 0.02) and −87.4 mm^3^ (tumor regression) after nab-paclitaxel plus BMS-754807 (*p* = 0.0001). In peritoneal dissemination model using AsPC-1 cells, median animal survival was increased compared to controls (21 days) after therapy with nab-paclitaxel (40 days, a 90% increase, *p* = 0.002), BMS-754807 (27 days, a 29% increase, *p* = 0.01) and nab-paclitaxel plus BMS-754807 (47 days, a 124% increase, *p* = 0.005), respectively. Decrease in proliferation and increase in apoptosis by nab-paclitaxel and BMS-754807 therapy correlated with their *in vivo* antitumor activity. *In vitro* analysis revealed that the addition of IC_25_ dose of BMS-754807 decreased the nab-paclitaxel IC_50_ of PDAC cell lines. BMS-754807 therapy decreased phospho-IGF-1R/IR and phospho-AKT expression, and increased cleavage of caspase-3 and PARP-1. These results support the potential of BMS-754807 in combination with nab-paclitaxel as an effective targeting option for pancreatic cancer therapy.

## INTRODUCTION

Pancreatic ductal adenocarcinoma (PDAC) is the fourth most common cause of cancer-related deaths in developed countries [1]. Surgical resection remains the best chance of long-term survival for patients with apparently localized PDAC. However, despite most advanced surgical techniques and antineoplastic combination treatments for advanced PDAC, the overall 5-year survival still remains low at 6% [2]. PDAC is expected to become the second-leading cause of cancer death by 2030, due to an increasing incidence and currently limited options to improve overall survival [3]. Less than 20% of PDAC patients are amenable to a curative surgical resection due to late clinical presentation, and early and aggressive local and metastatic progression [4]. Furthermore, even among these patients, postoperative recurrence remains commonplace [5, 6], leading to a 5-year survival at less than 20% [7]. Therefore, in recent years much attention has been directed toward improving systemic treatment options for PDAC. Gemcitabine, a nucleoside analog, has limited clinical benefits but it remained a standard treatment for locally advanced and metastatic PDAC since 1997 after producing a response rate of 5% and a median survival of 5.7 months [8]. FOLFIRINOX, a combination of chemotherapy agents (oxaliplatin, irinotecan, 5-FU/leucovorin), nearly doubled the median survival of PDAC patients (11.1 months compared with 6.8 months in the gemcitabine group), but this regimen is highly toxic with serious side effects [9]. Nab-paclitaxel (NPT), a water-soluble albumin-bound paclitaxel, has recently shown efficacy against advanced PDAC [10, 11], and nab-paclitaxel in combination with gemcitabine demonstrated 8.5 months median survival compared with 6.7 months after gemcitabine alone [12]. Considering these trial-based moderate improvements in PDAC prognosis, there is an urgent requirement for novel therapeutic strategies to improve overall patient survival.

PDAC progression is a multifactorial process, with overexpression of several growth factors and their receptors including epidermal growth factor (EGF), fibroblast growth factor (FGF), platelet-derived growth factor (PDGF), vascular endothelial growth factor (VEGF) and insulin-like growth factor (IGF), being associated with the highly aggressive nature of PDAC [13, 14]. The IGF signaling axis mainly consists of ligands IGF-1, IGF-2, insulin and IGF-1 receptor (IGF-1R), insulin receptor (IR) and hybrid receptors. IGF-1R is the primary mitogenic receptor of the IGF system that is overexpressed and has increased tyrosine kinase activity in several cancer types including PDAC [14–16]. Most of the tumorigenic effects of IGF signaling involves the binding of two ligands, IGF-1 and IGF-2, to IGF-1R, leading to the activation of several downstream signal transduction pathways including the phosphatidylinositol 3′-kinase (PI3K)/AKT and mitogen-activated protein kinase (MAPK) pathways [17]. Aberrant IGF signaling stimulates proliferation, differentiation, angiogenesis, metastasis, survival, and drug resistance in many cancers [18, 19], establishing this pathway as a generally promising therapeutic target. The IGF-1R pathway inhibition has been the subject of intensive anti-cancer drug discovery efforts and currently there are more than 30 active clinical trials evaluating anti-IGF-1R targeting agents, either alone or in various combinations (www.clinicaltrial.gov).

Three main strategies were employed to target the IGF pathway: monoclonal antibodies against IGF-1R, monoclonal antibodies against IGF-1R ligands (IGF-1 and IGF-2), and IGF-1R tyrosine kinase inhibitors. IGF-1R shares significant structural homology with IR, and targeting IGF pathway with IGF-1R inhibitor antibodies is sensible as it only blocks IGF-1R induced mitogenic signaling but is not affecting IR signaling, which could lead to dysregulation of glucose homeostasis [20, 21]. IGF-1R antibodies demonstrated clinical activity in phase 2 studies in small number of patients with select tumor types including Ewing sarcoma, thymoma and thymic carcinoma [22–24]. However, many clinical trials with IGF-1R inhibitors failed to show any significant clinical benefit including hepatocellular carcinoma [25], non-small cell lung cancer [26] and breast cancer [27]. In pancreatic cancer, the combination of gemcitabine and ganitumab, a monoclonal antibody against IGF-1R, showed a significant clinical response in a phase 2 randomized trial [28], but unfortunately this combination did not show any clinical benefit in phase 3 evaluation [29]. Recently, a clinical study demonstrated a correlation between IGF-1R signaling and tumor aggressiveness in PDAC patients [15], and increased exposure to ganitumab was found to be associated with improved overall survival and progression free survival in metastatic PDAC [30]. These findings demonstrate that IGF-1R remains a valid target for PDAC therapy.

Tyrosine kinase inhibitors can more indiscriminately regulate the kinase domain activity of all IGF system receptors as their primary sequence share 84% homology in the kinase domain with near complete conservation in the ATP binding pocket [31]. BMS-754807 (Supplemental Figure S1) is a potent and reversible inhibitor of IGF-1R/IR (IC_50_ 1.8 nM/1.7 nM), which also has more limited activity toward other kinases including Met, Aurora A/B, TrkA/B, Ron, Flt3, Lck, MK2, PKA and PKC [32, 33]. BMS-754807 has shown antitumor activity in a broad range of tumor types and it also enhanced antitumor response of other agents [33–36]. Recently, predictive biomarkers, including overexpression of IGF-1R, and KRAS and BRAF mutation status have been delineated for effectiveness of BMS-754807 [37]. In the present study, augmentation of response to nab-paclitaxel was evaluated by inhibition of IGF signaling in robust preclinical models of PDAC in an attempt to identify the efficacy of a novel therapeutic strategy.

## RESULTS

### Nab-paclitaxel and the IGF signaling inhibitor reduce the growth of PDAC xenografts

**Figure 1:**
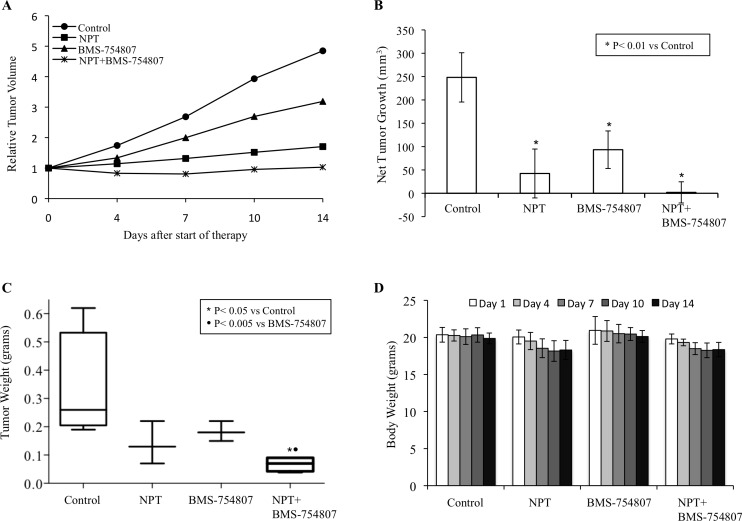
Antitumor activity of nab-paclitaxel and BMS-754807 in AsPC-1 tumor xenografts AsPC-1 cells were subcutaneously injected in nude mice and treated with nab-paclitaxel and BMS-754807 for 2 weeks. Tumor volume was measured twice a week using calipers. **A.** Relative tumor volume is calculated by dividing the tumor volume at any time by the tumor volume at the start of treatment. **B.** Net tumor growth was calculated by subtracting tumor volume on the first treatment day from that on the final day. **C.** Mean tumor weight was calculated from final day tumor weights in each group and is presented as a box plot. Box height denotes interquartile range; horizontal line within the box denotes median; and whiskers represent minimum and maximum values. **D.** Mouse body weight was measured twice a week and presented as bar chart for the 2-week therapy period. Data are representative of mean values ± standard deviation from 6-8 mice per group.

**Figure 2:**
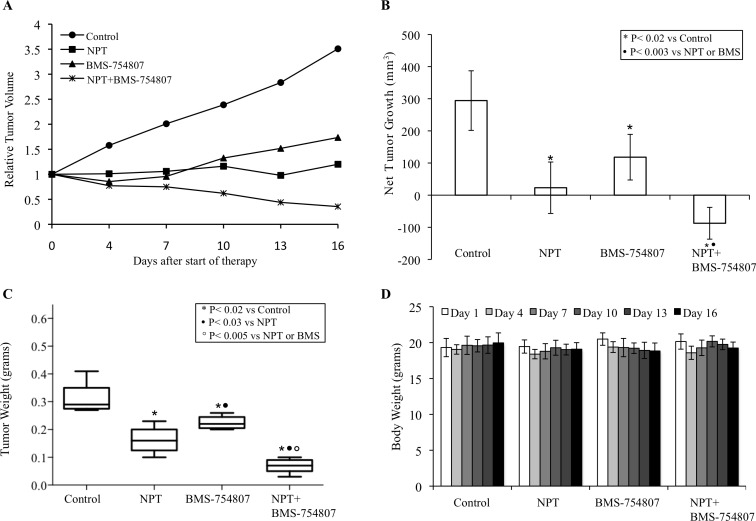
Antitumor activity of nab-paclitaxel and BMS-754807 in Panc-1 tumor xenografts Panc-1 cells were subcutaneously injected in nude mice and treated with nab-paclitaxel and BMS-754807 for 2 weeks. Tumor volume was measured twice a week using calipers. **A.** Relative tumor volume is calculated by dividing the tumor volume at any time by the tumor volume at the start of treatment. **B.** Net tumor growth was calculated by subtracting tumor volume on the first treatment day from that on the final day. **C.** Mean tumor weight was calculated from final day tumor weights in each group and is presented as a box plot. Box height denotes interquartile range; horizontal line within the box denotes median; and whiskers represent minimum and maximum values. **D.** Mouse body weight was measured twice a week and presented as bar chart for the 2-week therapy period. Data are representative of mean values ± standard deviation from 6-8 mice per group.

In the human subcutaneous PDAC xenografts, treatment of tumor-bearing mice with nab-paclitaxel and BMS-754807, an inhibitor of IGF signaling, caused significant antitumor effects as mono- and combination therapy of these agents demonstrated additive effects. In subcutaneous xenografts using AsPC-1 cells, nab-paclitaxel and BMS-754807 both caused a reduction in tumor growth while a combination of these two agents had additive effects (Figure 1A). Average net tumor growth, calculated by subtracting tumor volume on first therapy day from that on the last day, in different therapy groups was 248.3 mm^3^ in controls, 42.4 mm^3^ after nab-paclitaxel (*p* = 0.002), 93.3 mm^3^ after BMS-754807 (*p* = 0.01) and 1.9 mm^3^ after nab-paclitaxel plus BMS-754807 (*p* = 0.0002) (Figure 1B). At completion of therapy, mean tumor weight in different therapy groups was 0.33±0.19 g in controls, 0.14±0.08 g in nab-paclitaxel, 0.18±0.04 g in BMS-754807 and 0.07±0.03 g in nab-paclitaxel+BMS-754807 (Figure 1C). Furthermore, no significant change in total body weight was observed for those mice treated with nab-paclitaxel, BMS-754807 or combination (Figure 1D). In another subcutaneous PDAC xenograft experiment using Panc-1 cells, nab-paclitaxel and BMS-754807 treatment also caused a reduction in tumor growth with additive effects in combination (Figure 2A). Average net tumor growth in different therapy groups was 294.3 mm^3^ in controls, 23.1 mm^3^ after nab-paclitaxel (*p* = 0.002), 118.2 mm^3^ after BMS-754807 (*p* = 0.02) and −87.4 mm^3^ (tumor regression) after nab-paclitaxel plus BMS-754807 (*p* = 0.0001) (Figure 2B). Mean tumor weight in different therapy groups was: 0.30±0.06 g in controls, 0.16±0.05 g in nab-paclitaxel, 0.22±0.02 g in BMS-754807 and 0.07±0.03 g in nab-paclitaxel+BMS-754807 (Figure 2C). Also, no significant change in total body weight was observed for those mice treated with nab-paclitaxel, BMS-754807 or combination (Figure 2D).

### Nab-paclitaxel and the IGF signaling inhibitor improve animal survival

**Figure 3:**
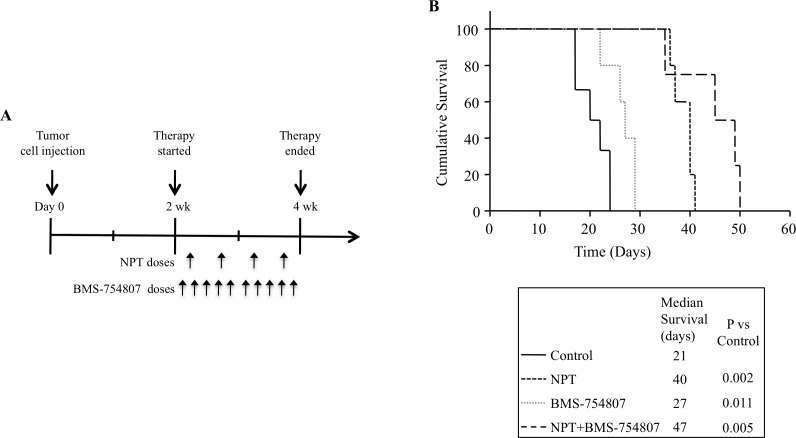
Improvement in animal survival by nab-paclitaxel and BMS-754807 **A.** Schematic representation of experimental procedure. AsPC-1 cells (0.75 × 10^6^) were injected intraperitoneally in NOD/SCID mice and treatment started after 2 weeks with nab-paclitaxel and BMS-754807 for 2 weeks. **B.** The curve represents the animal survival time from the beginning of therapy. Statistical group differences in survival time were calculated using logrank testing (GraphPad Prism 6.0).

In the human PDAC peritoneal dissemination model using AsPC-1 cells in NOD/SCID mice, nab-paclitaxel and BMS-754807 therapy was started two weeks after tumor cell injection and was continued for the subsequent two weeks (Figure 3A). Animal survival in different therapy groups, calculated from the start of therapy, was as follows: controls (21 days), nab-paclitaxel (40 days, a 90% increase compared with controls, *p* = 0.002), BMS-754807 (27 days, a 29% increase compared with controls, *p* = 0.01) and nab-paclitaxel+BMS-754807 (47 days, a 124% increase compared with control, *p* = 0.005) (Figure 3B). There was no significant change in mouse body weight during two week therapy period in all groups, indicating that there was no significant drug-related toxicity in monotherapy or combination therapy groups.

### Effects of nab-paclitaxel and the IGF signaling inhibitor on intratumoral proliferation and apoptosis

Possible mechanisms for the antitumor activities of nab-paclitaxel and BMS-754807 were investigated by IHC analysis of tumor tissues obtained from subcutaneous xenograft studies after completion of two weeks of therapy.

**Figure 4:**
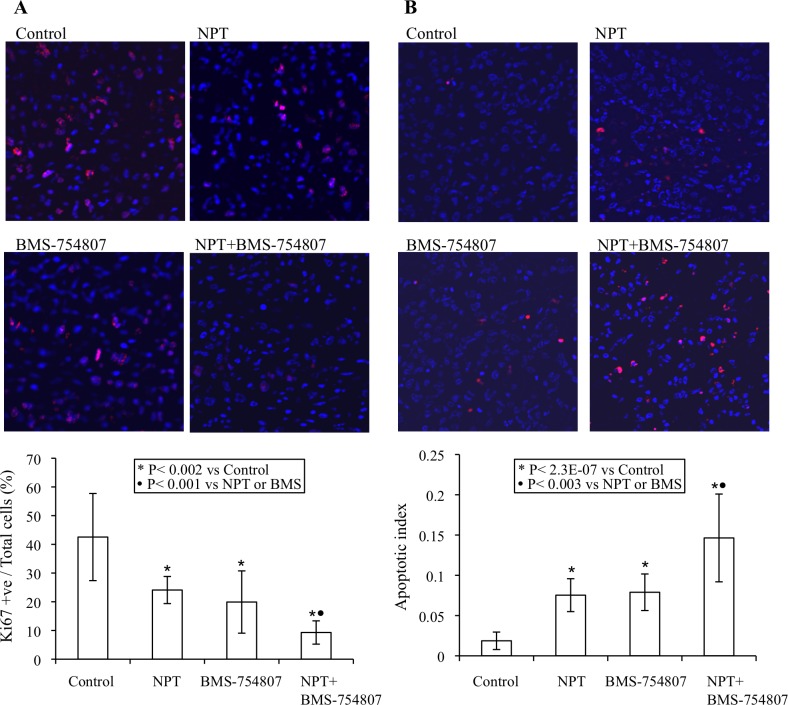
Mechanisms of antitumor activity of nab-paclitaxel and BMS-754807 Nude mice were subcutaneously injected with AsPC-1 cells and treated with nab-paclitaxel and BMS-754807 for 2 weeks. **A.** Intratumoral proliferation was measured by immunostaining tissue sections for Ki67 nuclear antigen. Ki67-positive cells were counted in five different high power fields. **B.** Intratumoral apoptosis was measured by staining tumor tissue section with TUNEL procedure. TUNEL-positive apoptotic cells were counted in five different high power fields. For both immunostaining experiments, slides were photographed under a fluorescent microscope and the data are expressed as the mean ± standard deviation.

Intratumoral proliferative activity was analyzed by Ki67 staining and this analysis demonstrated that nab-paclitaxel and BMS-754807 inhibited intratumoral cell proliferation and combination therapy group generated additive effects. Different therapy groups showed the following intratumoral proliferative indices, calculated by percentage of Ki67-positive cells over total number of cells per HPF: controls (42.5±15.2), nab-paclitaxel (24.1±4.7), BMS-754807 (19.9±10.8) and nab-paclitaxel+BMS-754807 (9.3+4.1) (Figure 4A).

Intratumoral apoptosis, measured by TUNEL assay, revealed that the nab-paclitaxel and BMS-754807 monotherapy increased intratumoral apoptosis that was further increased in the combination therapy group. The apoptotic index, calculated by number of TUNEL-positive cells over total number of cells per HPF, in these therapy groups: controls (0.02±.01), nab-paclitaxel (0.08±0.02), BMS-754807 (0.08±0.02) and nab-paclitaxel+BMS-754807 (0.15±0.05) (Figure 4B).

**Figure 5:**
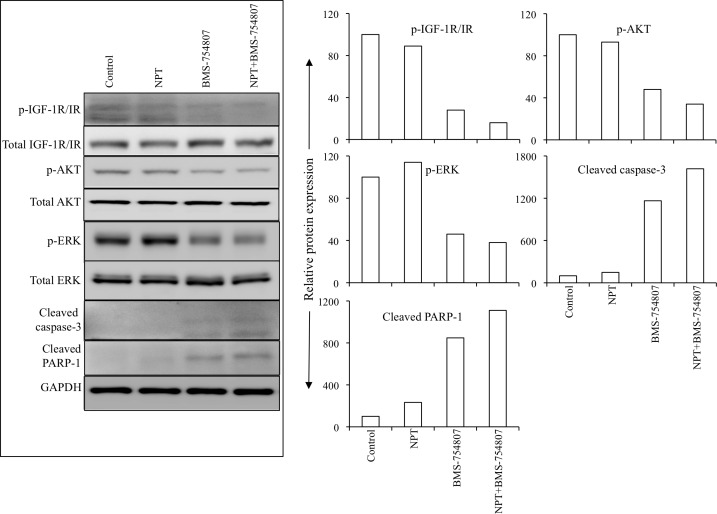
Nab-paclitaxel and BMS-754807 effects on the IGF signaling pathways and apoptosis-related proteins *in vivo* Tumor lysates were prepared from tumor tissues obtained from AsPC-1 tumor-bearing mice and were analyzed by immunoblotting. Data are representative of pooled lysates obtained from tumor sections of at least 5 mice in each therapy group. The intensity of bands was quantitated by densitometry and is represented in the bar graph after normalizing values with corresponding total protein expression or GAPDH expression.

Additional mechanisms of *in vivo* antitumor activity of nab-paclitaxel and BMS-754807 were examined by analyzing intratumoral expression of their molecular targets by Western blot analysis. BMS-754807 therapy caused a significant decrease in the expression of phospho-IGF-1R/IR, and decreased the expression of the downstream signaling proteins phospho-AKT and phospho-ERK. Furthermore, BMS-754807 treatment caused a concomitant increase in the apoptosis marker proteins cleaved caspase-3 and cleaved PARP-1 (Figure 5).

### Effects of nab-paclitaxel and the IGF signaling inhibitor on PDAC cell proliferation and cellular targets *in vitro*

**Figure 6:**
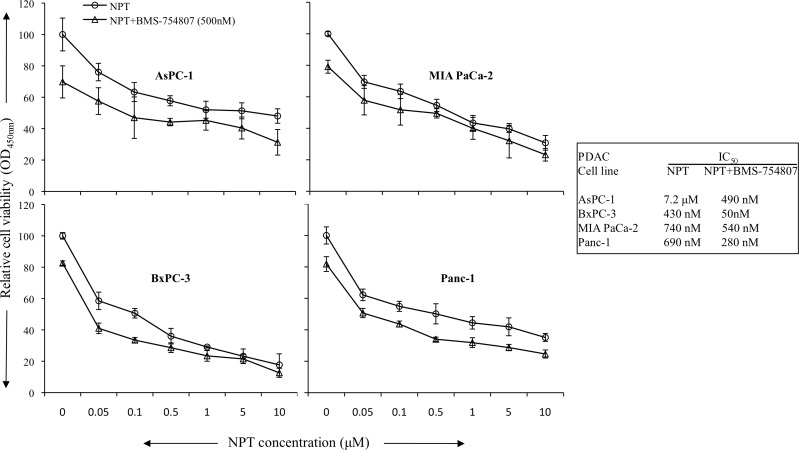
Addition of BMS-754807 had additive effects in combination with nab-paclitaxel to inhibit *in vitro* cell proliferation of PDAC cells PDAC cells were plated on 96-well plates and treated with nab-paclitaxel (0 to 10 μM) as depicted through the open circle line. Another group of cells were treated with IC_25_ dose of BMS-754807 (500 μM) and nab-paclitaxel as depicted through the open triangle line. After 72 hours, 10 μl WST-1 reagent was added in each well and incubated for 2 additional hours. The absorbance at 450 nm was measured using a microplate reader. The resulting number of viable cells was calculated by measuring absorbance of color produced in each well. Data are the mean ± SD of triplicate determinations.

**Figure 7:**
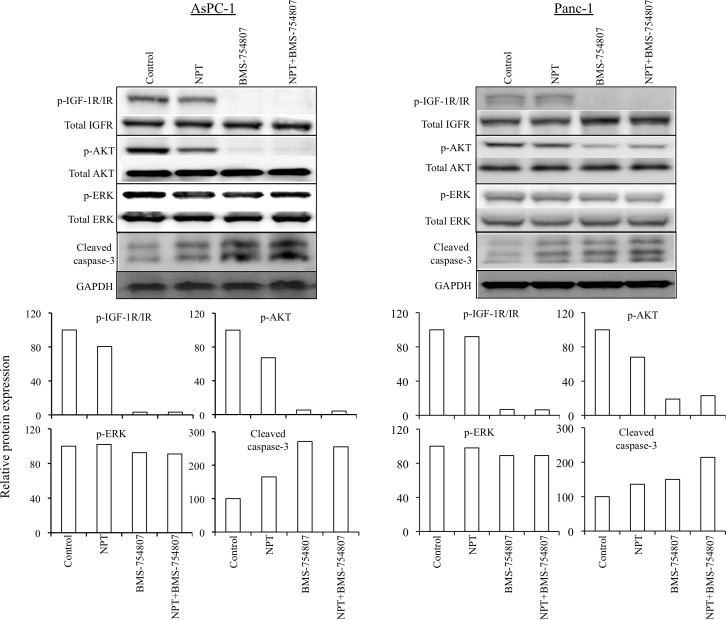
Nab-paclitaxel and BMS-754807 effects on the IGF signaling pathway and apoptosis-related proteins A sub-confluent monolayer of human PDAC cells AsPC-1 and Panc-1 was treated with nab-paclitaxel (10 μM) and BMS-754807 (10 μM), either alone or in combination for 16 hours. Total cell extracts were analyzed by immunoblotting. The intensity of bands was quantitated by densitometry and is represented in the bar graph after normalizing values with corresponding total protein expression or GAPDH expression. Data are representative of two independent experiments with similar results.

The antitumor mechanism of nab-paclitaxel and BMS-754807 was evaluated by analyzing *in vitro* cell viability of human PDAC epithelial cells with different mutation status. Both, nab-paclitaxel and BMS-754807, inhibited PDAC cells proliferation. The combination treatment benefits of nab-paclitaxel and BMS-754807 were observed by adding the IC_25_ dose of BMS-754807 with increasing doses of nab-paclitaxel. Addition of BMS-754807 decreased the nab-paclitaxel IC_50_ from 7.2 μM to 490 nM for AsPC-1, 430 nM to 50 nM for BxPC-3, 740 nM to 540 nM for MIA PaCa-2, and 690 nM to 280 nM for Panc-1 cell lines (Figure 6). Immunoblot analysis to determine the effect of the IGF signaling inhibitor on the IGF/receptor axis in AsPC-1 and Panc-1 cells, revealed that BMS-754807 blocked the expression of phospho-IGF-1R/IR. BMS-754807 treatment also decreased the expression of downstream signaling protein phospho-AKT but there was no significant effect on phospho-ERK expression. Nab-paclitaxel and BMS-754807 induced the expression of apoptosis-related cleaved caspase-3 protein, while combination treatment generated an additive effect (Figure 7).

## DISCUSSION

Activation of the IGF signaling pathway appears to be an important mechanism for progression of several malignancies. IGF-1R overexpression has been correlated with tumor aggressiveness [43–45] while disruption of IGF-1R activation inhibited growth and motility of a wide range of cancers [46]. Additionally, the blockage of IGF-1R signaling has been shown to enhance chemotherapy response in preclinical studies of several cancer types including pancreatic and non small-cell lung cancers as well as Ewing's sarcoma [43, 47, 48]. Since IGF-1R and IR are highly homologous, antagonist development was initially focused towards monoclonal antibodies that selectively target IGF-1R and not affect IR signaling, which could lead to dysregulation of glucose homeostasis [21]. Initial clinical studies with IGF-1R antibodies showed some responses in monotherapy and in combination with cytotoxic chemotherapy [49]. However, patients receiving antibody therapy have hyperglycemia indicating the potential of undesirable metabolic side effects of such therapy [50]. Several reports showed the involvement of stimulation of IR by insulin or IGF-2 in cancer cell progression [51, 52] suggesting that IGF-1R and IR both are therapeutic targets and inhibition of both receptors may be required for optimal tumor growth inhibition. In PDAC specifically, the IGF signaling activation has also been shown to play a major role in tumor progression [14–16]. In addition, a recent study demonstrated a correlation between IGF-1R signaling and tumor aggressiveness in PDAC patients [15]. Based on these aspects and given the recent clinical progress in PDAC cytotoxic therapy, our present study thus explored the effects of BMS-754807, a small molecule inhibitor of both the receptors IGF-1R/IR, in combination with the recently approved and more effective chemotherapeutic agent nab-paclitaxel.

In multiple PDAC epithelial cells, we observed a differential expression of phosphorylated form of IGF-1R/IR (Supplemental Figure S2), which is consistent with a previously published study [14], indicating a possible involvement of aberrant IGF signaling in PDAC progression. The IGF signaling inhibitor BMS-754807 has shown antitumor activity in multiple tumor models including epithelial, mesenchymal and hematopoietic cancer cells [33]. We observed the effect of BMS-754807 in subcutaneous xenografts using two aggressive PDAC cell lines, AsPC-1 and Panc-1. In these models, BMS-754807 inhibited tumor growth as a single agent and more importantly, caused additive effects in combination with nab-paclitaxel. Analysis of intratumoral proliferation and apoptosis appear to be correlated with tumor growth inhibition data. Evaluation of target proteins in tumor lysates showed that BMS-754807 treatment decreased levels of the activated form of IGF-1R/IR and downstream effectors AKT and ERK, and induced levels of the apoptosis related proteins cleaved caspase-3 and cleaved PARP-1, indicating that the BMS-754807 therapy is indeed managing to sufficiently affect these targets within the local tumor model. Furthermore, BMS-754807 and nab-paclitaxel demonstrated a significant additive response to improve animal survival as evaluated in a peritoneal dissemination model using AsPC-1 cells which is highly reproducible, well characterized and closely resembles the metastatic progression pattern of the clinical disease. In the present study, we have not analyzed the effect of dosing schedule of the drugs where nab-paclitaxel was given prior to BMS-754807 to evaluate nab-paclitaxel induced tumor permeability. However, in the present study dosing schedule, nab-paclitaxel (twice per week) and BMS-754807 (five times per week) were given together on day 1; therefore, for all other doses of BMS-754807, nab-paclitaxel effects on tumor permeability might already be in place.

BMS-754807 inhibited the proliferation of all PDAC cells tested; importantly, in all cell lines addition of IC_25_ dose of BMS-754807 decreased the IC_50_ values of nab-paclitaxel. Additionally, in AsPC-1 and Panc-1 PDAC epithelial cells, BMS-754807 treatment caused a decrease in phosphorylated IGF-1R/IR and its downstream signaling protein phospho-AKT, no significant change in phospho-ERK, but increased cleavage of apoptosis related caspase-3 protein. A possible explanation for BMS-754807 to block ERK phosphorylation in AsPC-1 tumor lysates but not in AsPC-1 or Panc-1 cell lines is the significant activity of stromal cells within tumors, in which ERK-phosphorylation has been shown to be blocked by BMS-754807 (own data, not shown here). A combination of these molecular signaling changes are thus likely operational in the BMS-754807 induced inhibition in cell proliferation and induction in apoptosis, and appear to represent good markers of *in vivo* activity testing and for clinical validation.

Active PDAC progression involves multiple distinct mechanisms including induction in cancer cell proliferation, migration, differentiation, angiogenesis and inhibition of cancer or stromal cell apoptosis. Previous studies in our lab have shown the benefits of targeting multiple pathways in different cellular compartments including epithelial, vascular and stromal elements for PDAC treatment [39, 40, 42, 53]. Nab-paclitaxel itself has antiproliferative and proapoptotic effects on tumor cells, endothelial cells and fibroblasts. The exact molecular mechanisms for the enhancement in antitumor activity of nab-paclitaxel by BMS-754807 addition is not completely clear, but additive benefits are likely caused by augmentation of antiproliferative and proapoptotic activities and possibly not just restricted to the tumor cells of epithelial origin. Because of the multifactorial nature of PDAC and limited effectiveness of standard chemotherapy agents, combination therapies targeting multiple pathways represent sensible strategies for clinical evaluation. Based on our findings the combination of nab-paclitaxel and BMS-754807 is a reasonable clinical trial option for phase II evaluation after establishing toxicity of this combination.

Of note, most of the negative clinical trials of the IGF signaling inhibitors used monoclonal antibodies specifically targeting IGF-1R and not IR [25–27]. This could have potentially provided escape mechanisms for insulin and IGF-2 signaling that have been implicated in cancer cell progression [51, 52], leading to limited clinical response. Therefore, simultaneous inhibition of IGF-1R and IR by BMS-754807 may confer a better antitumor effect. Results from our prior work with BMS-754807 and gemcitabine as single cytotoxic agent in preclinical PDAC therapy would certainly support this broader targeting approach [36]. PDAC is histologically characterized by a dense desmoplastic reaction surrounding malignant epithelial cells that plays a critical role in tumor progression, invasion, metastasis and drug-resistance by providing a mechanical barrier. Nab-paclitaxel has been proposed to disrupt the PDAC stromal architecture, causing increased perfusion and drug-delivery [12]. Our laboratory has shown that the nab-paclitaxel is the most effective single agent cytotoxic agent in comparison with gemcitabine or docetaxel [10]. In addition, nab-paclitaxel combination with targeted therapies has been shown to efficiently improve antitumor response [11, 54]. Therefore, the application of combination therapy benefits of nab-paclitaxel with BMS-754807 seems particularly logical and plausible for advanced PDAC.

Since BMS-754807 inhibits both IGF-1R and IR signaling, dysregulation of glucose homeostasis is an important concern, and therefore the advantage of its antitumor effects might have to be balanced with the potential for metabolic side effects. In our studies, there was no apparent *in vivo* toxicity during a 2-week treatment course, however, toxicity for long-term inhibition of the IGF-1R and IR functions remains to be elucidated. A previous study reported only a short-term increase in serum glucose or insulin levels at doses up to 12.5 mg/kg, with an average weight change of −1.5 grams at 25 mg/kg, but no mortality [33]. In our study, no significant change in mouse body weight was observed by BMS-754807 therapy, but blood glucose measurements were not obtained. In conclusion, our study indicates that the combined IGF-1R/IR signaling inhibitor, BMS-754807, can mediate mechanism-specific antitumor activity in experimental PDAC, and significantly improves nab-paclitaxel response. These findings corroborate the rationale of blocking multiple pathways of the IGF and insulin signaling, and support the potential of BMS-754807 as targeting agent for clinical PDAC therapy.

## MATERIALS AND METHODS

### Cell culture and reagents

Human PDAC cell lines (AsPC-1, BxPC-3, Mia PaCa-2 and Panc-1) were purchased from the American Type Culture Collection (ATCC, Rockville, MD). Cell lines were tested and authenticated by ATCC. Cells were initially grown and multiple aliquots were cryopreserved. All the cell lines were used within 6 months after reexpansion in culture. Cells were cultured in DMEM or RPMI 1640 medium (Sigma Chemical Co. St. Louis, MO) containing 10% FBS and maintained at 37°C in a humidified incubator with 5% CO_2_ and 95% air. BMS-754807 was purchased from Active Biochem (Maplewood, NJ). The cell proliferation reagent WST-1 was purchased from Roche Diagnostic Corporation (Indianapolis, IN).

### Western blot analysis

PDAC cells were treated with nab-paclitaxel (10 μM) and BMS-754807 (10 μM), and lysed after 16 hours to prepare cell lysate for Western blotting. Tumor lysates were prepared by snap freezing tumor tissues in liquid nitrogen and stored at −80°C. These tumor tissues were suspended in lysis buffer and homogenized using the Bullet Blender Homogenizer (Next Generation, Averill Park, NY), and extracts were sonicated on ice. Proteins in supernatants were separated by SDS-PAGE and transferred to PVDF membranes (Bio-Rad, Hercules, CA). The membranes were incubated overnight at 4°C with the following antibodies: total IGF-1R, phospho-IGF-1R (Tyr1135/1136)/IR (Tyr1150/1151; #3024), total AKT, phospho-AKT (Ser473), total ERK1/2, phospho-ERK1/2 (Thr202/Tyr204), cleaved caspase-3, cleaved PARP-1 and GAPDH (Cell Signaling Technology, Beverly, MA). The membranes were then incubated with the corresponding HRP-conjugated secondary antibodies (Pierce Biotechnologies, Santa Cruz, CA) for 1 to 2 hour. Specific protein bands were visualized using the enhanced chemiluminescence reagent (SignalFire, Cell Signaling) with Image360 system and quantitated by densitometry.

### Cell viability assay

Cell viability of PDAC lines was evaluated by the colorimetric WST-1 assay as previously described [38]. Briefly, PDAC cells (4,000 cells per well) were plated in a 96-well plate in regular growth medium. After 16 hours the medium was replaced with 2% FBS containing medium and the cells were treated with nab-paclitaxel (50 nM to 10 μM) and IC_25_ dose of BMS-754807 (500 nM). After 72 hours, 10 μl WST-1 reagent was added in each well followed by additional incubation for 2 hours. The absorbance was measured at 450 nm using a microplate reader.

### Animal studies

All animals were housed in pathogen-free facility with access to food and water *ad libitum*. Animal experiments were performed in accordance with the Institutional Animal Care and Use Committee (IACUC) at the Indiana University School of Medicine (South Bend, IN). Female nonobese diabetic/severe combined immunodeficient (NOD/SCID) mice (4 to 6 weeks old) were subcutaneously injected with AsPC-1 cells (0.75 × 10^6^) or Panc-1 cells (10 × 10^6^) as previously described [11]. Two weeks after tumor cell injection, all mice had measurable tumor. Mice were then randomized (*n* = 6 to 8 per group) to receive PBS (control), nab-paclitaxel (10 mg/kg, twice a week) and (BMS-754807 (25 mg/kg in 100 ml PBS, 5 times a week) *via* intraperitoneal injection for 2 weeks. The tumor size was measured twice weekly, and tumor volume (V) was calculated using the formula V = ½ (Length x Width^2^). Mice were euthanized after completion of treatment, tumors were dissected and processed for histological, immunohistochemical and Western blot analysis.

Animal survival studies were performed using female NOD/SCID mice (4-6 weeks of age) as previously described [39]. Briefly, the mice were intraperitoneally injected with AsPC-1 (0.75×10^6^) cells and two weeks after tumor cell injection, mice were randomized (*n* = 6 to 8 per group) to receive PBS (control), nab-paclitaxel (10 mg/kg, twice a week) or BMS-754807 (25 mg/kg in 100 ml PBS, 5 times a week) *via* IP injection for two weeks. Animals were euthanized when moribund according to predefined criteria [40, 41]. Animal survival was evaluated from the first day of treatment until death.

### Immunohistochemistry and immunofluorescence

Standard immunohistochemistry protocol was followed to stain the tumor tissue samples, as previously described [42]. Briefly, tumor tissue samples were fixed in 4% paraformaldehyde, embedded in paraffin, tissue sections were cut (5 μm), deparaffinized and rehydrated. The tissue sections were incubated with Ki67 antibody (1:200 dilution) followed by incubation with Cy3 (1:200 dilution) secondary antibody. Slides were mounted using mounting solution containing 4′,6-diamidino-2-phenylindole (DAPI) (Invitrogen, Carlsbad, CA). Intratumoral proliferative index was evaluated by calculating the Ki67-positive cells from five different high-power fields (HPF) in a blinded manner. Intratumoral apoptotic activity was evaluated by staining tissue sections with “Apoptag Apoptosis Detection Kit” according to the manufacturer's (Millipore, Temecula, CA) instructions. Fluorescence microscopy was used to detect fluorescent signals using the IX81 Olympus microscope equipped with a Hamamatsu Orca digital camera (Hamamatsu Corporation, Bridgewater, NJ) and a DSU spinning confocal unit using Slidebook software (Intelligent Imaging Innovations, Philadelphia, PA).

### Statistical analysis

Statistical analysis for *in vivo* tumor growth studies was performed by one-way ANOVA for multiple group comparison and Student's t-test for the individual group comparison. Survival study statistics were performed using logrank group comparison (GraphPad Prism 6.0). P values less than 0.05 were considered statistically significant. *In vitro* cell proliferation data are expressed as mean ± standard deviation. Statistical significance was analyzed by the two-tailed Student's t-test using GraphPad Prism 6.0 Software (GraphPad Software, San Diego, CA) for individual group comparisons.

## SUPPLEMENTARY MATERIAL


