# Supplementary material for: Augmentation of response to nab-paclitaxel by inhibition of insulin-like growth factor (IGF) signaling in preclinical pancreatic cancer models
Source: Oncotarget. 2016 Apr 26;7(30):46988–7001. doi: 10.18632/oncotarget.9029 (PMC5216918; doi:10.18632/oncotarget.9029)
Supplement: Supplementary file 1 [file oncotarget-07-46988-s001.pdf]

Augmentation of response to nab-paclitaxel by inhibition of insulin-like growth factor (IGF) signaling in preclinical pancreatic cancer models

Supplementary Material

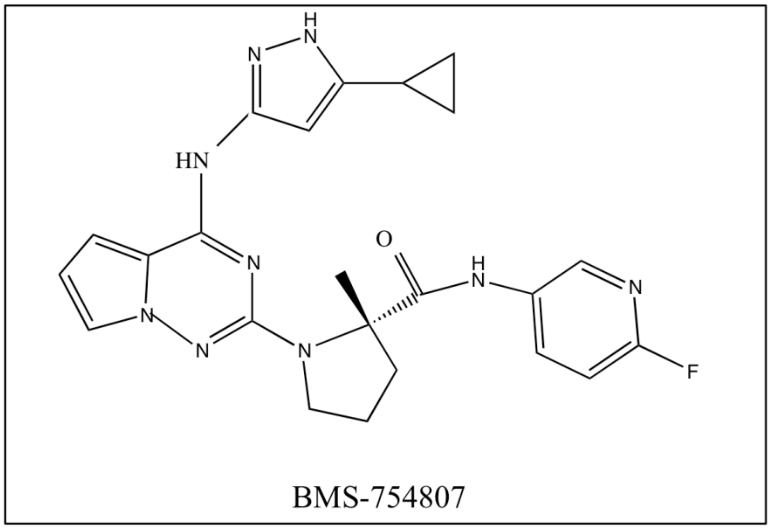

Supplemental Figure S1: Chemical structure of BMS-754807. Molecular weight: 461.49

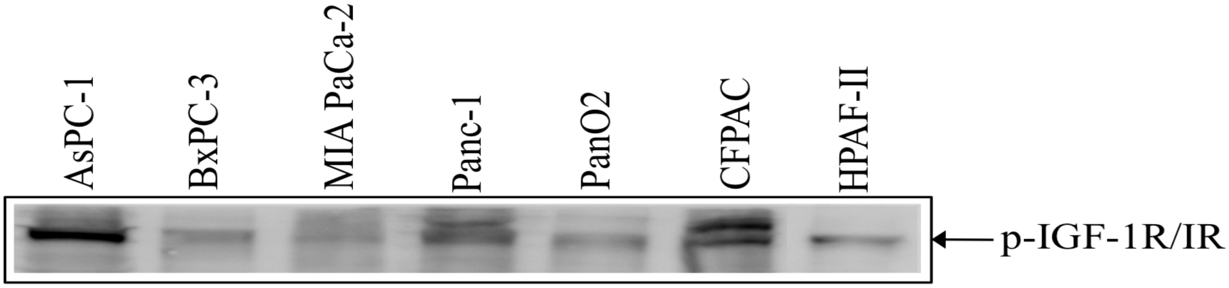

Supplemental Figure S2: Expression of phospho-IGF-1R/IR in PDAC cell lines. Total cell extracts of PDAC cells were analyzed by immunoblotting for phospho-IGF-1R/IR protein.
